# Comparison of Knowledge, Attitude, and Practice among Communities Living in Hotspot and Non-Hotspot Areas of Dengue in Selangor, Malaysia

**DOI:** 10.3390/tropicalmed4010037

**Published:** 2019-02-15

**Authors:** Nurul Akmar Ghani, Shamarina Shohaimi, Alvin Kah-Wei Hee, Hui-Yee Chee, Oguntade Emmanuel, Lamidi Sarumoh Alaba Ajibola

**Affiliations:** 1 Department of Biology, Faculty of Science, Universiti Putra Malaysia, 43400 Serdang, Selangor, Malaysia; alvinhee@upm.edu.my; 2 Institute for Mathematical Research, Universiti Putra Malaysia, 43400 Serdang, Selangor, Malaysia; oguntadeemmanuel2015@gmail.com; 3 Department of Medical Microbiology and Parasitology, Faculty of Medicine and Health Sciences, Universiti Putra Malaysia, 43400 Serdang, Selangor, Malaysia; cheehy@upm.edu.my; 4 Department of Mathematics, Faculty of Science, Universiti Putra Malaysia, 43400 Serdang, Selangor, Malaysia; lalabaajibolasarulam@gmail.com

**Keywords:** dengue, knowledge attitude practice (KAP), dengue incidence, dengue hotspot

## Abstract

Background: Dengue has affected more than one-third of the world population and Malaysia has recorded an increase in the number of dengue cases since 2012. Selangor state recorded the highest number of dengue cases in Malaysia. Most of the dengue infections occur among people living in hotspot areas of dengue. This study aims to compare Knowledge, Attitude, and Practice among communities living in hotspot and non-hotspot dengue areas. Method: Communities living in 20 hotspot and 20 non-hotspot areas in Selangor were chosen in this study where 406 participants were randomly selected to answer questionnaires distributed at their housing areas. Total marks of each categories were compared using *t*-test. Result: Results show that there were significant mean differences in marks in Knowledge (*p* value: 0.003; 15.41 vs. 14.55) and Attitude (*p* value: < 0.001; 11.41 vs. 10.33), but not Practice (*p* value 0.101; 10.83 vs. 10.47) categories between communities of non-hotspot and hotspot areas. After considering two confounding variables which are education level and household income, different mean marks are found to be significant in Knowledge when education level acts as a covariate and Attitude when both act as covariates. Conclusion: Overall results show that people living in non-hotspot areas had better knowledge and attitude than people living in hotspot areas, but no difference was found in practice. This suggests that public health education should be done more frequently with people with a low education background and low household income, especially in hotspot areas to fight dengue outbreak and make dengue cases decrease effectively.

## 1. Introduction

Dengue infections are causing the biggest number of deaths of all arbovirus diseases globally. More than one-third of the worldwide population are affected by dengue [1]. Dengue fever is characterized by fever, muscle and joint paints, rash, nausea, vomiting, and headache [2]. Asia continues to bear the burden of high dengue cases and Southeast Asia, the tropical and warmer part of Asia, reported higher cases, mortality and morbidity rates of dengue annually [3]. Dengue virus is spread by *Aedes* mosquitoes, and the most common vectors are *Aedes aegypti*, followed by *Aedes albopictus*, *Aedes polynesiensis*, and *Aedes sutellaris* [2].

As a country located in Southeast Asia, Malaysia also experiences a high number of dengue cases which fluctuates from one year to another. For example, in 2013 the number of dengue cases was 43,436 cases, and the number tremendously increased in 2014 and 2015 to 108,698 and 120,836, respectively. The number of dengue cases in Malaysia slightly decreased in 2016 with 101,357 cases. However, it was still high compared to the years before 2014 [4]. Among all states in Malaysia, Selangor recorded the highest number of dengue cases with approximately 50% of Malaysian dengue cases occurring in this state, with a total number of cases 62,867 and 51,652 in 2015 and 2016, respectively [5].

Selangor is a state with a high population density in Malaysia compared to other states, and the behavior of its citizens has a high influence on dengue spread and transmission since household-level plays an important part in mosquito control to prevent the disease [5,6]. The use of a Knowledge, Attitude, and Practice (KAP) questionnaire has helped researchers significantly in investigating the level of KAP of particular residents in a location inrespect to the disease, besides assisting researchers in examining the factors contributing to the spread of a particular disease [7]. A KAP survey also provides a format to evaluate the effectiveness of current health programs [8].

The survey consists of three categories; Knowledge, Attitude, and Practice, in which every category is separated and given marks respectively before the overall mark is calculated. The first part represents Knowledge which tests people’s knowledge of that particular disease. It provides an overview of the level of knowledge a certain population has about the disease. The second part is Attitude which accesses people’s attitude towards the disease. They are asked about their view of the disease and how they would react if they or anyone around them was infected by the disease. The third part is Practice where participants are asked about what their daily routines are to prevent the disease. When it comes to doing studies related to infectious disease, the measure of all survey parts helps to estimate the extent of the real situation among the current population as well as confirming any theories of infectious disease spread related to humans. From the survey, researcher know the type of information about the disease that population already knows, so the next step is to focus on information not yet disseminate among them, such as the ability to change health-related behavior which is an attitude that is highly correlated with knowledge. In an infectious disease which is environmentally associated, human involvement is very highly correlated to prevent or boost the spread of the disease [7].

In Malaysia, a dengue outbreak happens when four or more cases are reported in the same housing area within a week. A hotspot dengue area is characterized by an area which has a dengue outbreak for more than 30 days, while a non-hotspot area is an area free from dengue outbreak for more than 30 days. [9] This study aims to compare the level of Knowledge and Attitude between communities living in hotspot and non-hotspot areas of dengue outbreak.

## 2. Materials and Methods

A cross-sectional survey using a self-administered questionnaire was conducted from November 2015 to October 2016 in 40 selected hotspot and non-hotspot areas in Selangor. Systematic random sampling was applied during data collection where 1 respondent in every 3 houses that researchers passed by was chosen to answer the questionnaire, with their consent. Those who could read and were aged 17 years old above were included in this study. The rationale of choosing locations in this study was so that the results would have both comparable numbers of respondents from the hotspot and non-hotspot areas answering the questionnaire.

The sample size was calculated using A-priori Sample Size Calculator where 400 minimum samples were needed for this study. The real sample size was 406 respondents, where 205 came from hotspot areas, and the rest were respondents from non-hotspot areas. The questionnaire used in this study was the improved version of the questionnaire validated by Mohammad Nasir et al. [8]. A pilot study (n = 30) was done to ensure the questionnaire carried effective, efficient, reliable, and valid data. The questionnaire was in the Malay language, the national language of Malaysia.

The questionnaire comprized a few sections; Demographic data and characteristics of respondents, Knowledge and Attitude. It contained 16 items for Knowledge and 15 items for Attitude. The knowledge category had Yes/No/Not sure response, and Attitude had Agree/Disagree response. A correct and positive answer was given mark 1 while wrong and negative answer got no mark. Total marks of correct answers were then calculated for each category.

Frequency and percentage were analyzed using descriptive statistic, while the Student’s *t*-test was used to compare the significant difference of marks of each category and statistical significance was set at *p* < 0.05.

Ethical Approval: Ministry of Health, Malaysia via National Medical Research Registry (reference approval number: NMRR-15-2133-28404 (IIR)).

## 3. Results

Four hundred and six respondents answered the questionnaire, and all were included in the analysis. Table 1 summarizes the demographic characteristics of the respondents.

Table 1 shows the demographic characteristics of respondents for the questionnaire. Most of them were female, Malay, had a household income of RM5000 or less, had an education level of SPM or less, and most of them were not infected by dengue fever.

Table 2 illustrates the respondents’ knowledge of dengue fever. Most people living in hotspot and non-hotspot areas knew which mosquito species cause dengue fever and understood that a person can get dengue fever more than once. However, both respondents in hotspot and non-hotspot areas assumed dengue fever as non-infectious disease. More than half of respondents from both areas thought that dengue only happens during the rainy season. For dengue symptoms, respondents from both areas showed little difference in their knowledge. A higher percentage of respondents living in non-hotspot areas believed that *Aedes* does not only spread virus during the day but *Aedes* also does not reproduce in dirty water and were sure that larvae killer can help kill *Aedes* larvae. Respondents from both areas showed equivalent knowledge on dengue treatment and were confident that insect repellent can kill adult mosquitoes. 

In conclusion, both people living in hotspot and non-hotspot areas showed comparable knowledge about dengue fever with people living in non-hotspot areas scored slightly higher marks of knowledge in total.

Table 3 shows the respondents’ attitude towards dengue. More respondents from non-hotspot areas believed that dengue can be prevented, fogging is not enough to avoid dengue, and agreed that a person can get dengue twice, compared to respondents from hotspot areas. More respondents from hotspot areas felt afraid if they are infected by dengue. A higher percentage of respondents from non-hotspot areas disagreed that killing *Aedes* breeding sites was a waste of time. All but two respondents from non-hotspot areas believed that they play important roles to fight dengue outbreak, compared to only 178 out of 205 respondents from hotspot areas. Overall, people living in non-hotspot dengue areas demonstrated a more positive attitude towards dengue fever compared to people living in hotspot areas.

Table 4 shows respondents’ practice towards dengue vector control. People living in hotspot and non-hotspot dengue outbreak areas demonstrated little difference in practice in controlling *Aedes* mosquitoes. More respondents in non-hotspot areas reported that they checked for rubbish that may clog their drains, put garbage in bins, changed the water in flower vase more often and changed the water in containers of their garden more frequently. Meanwhile, other practice questions illustrated a comparable number of respondents from both areas.

Table 5 shows the result of *t*-test analysis comparing mean marks of knowledge, attitude, and practice between hotspot and non-hotspot communities. It illustrates that non-hotspot communities show higher mean marks in knowledge and attitude categories compared to hotspot communities, but no significant difference found in the mean mark of practice category between both communities. It reveals that communities living in non-hotspot areas had better knowledge and a better attitude about dengue fever compared to communities living in hotspot areas, as *p* value for both Knowledge and Attitude categories were less than 0.05. However, non-hotspot and hotspot communities show no difference in practice which demonstrates to us that their daily routines do not differ much. This elucidates that even though non-hotspot communities shows better knowledge and attitude about dengue, it does not make them better in practicing their knowledge into their daily life.

Table 6 shows the result of Analysis of Covariances where education level and household income are considered in the analysis, whether they have an effect or not on knowledge, attitude, and practice of participants. For knowledge results, the mean difference shows a significant *p* value when education level acts as the confounding factor, which means the variance in knowledge marks is 1.1% explained by education level. However, when household income acts as a confounding factor, the mean of knowledge marks between hotspot and non-hotspot communities is not significantly different. This suggests that their knowledge of dengue within the same range of household income does not differ despite living in different areas of dengue outbreak. For attitude marks, the mean marks are still significantly different when both education level and household income act as confounding factors, which means the variances of attitude marks are explained by education level and household income factors at 5% and 3.8%, respectively. Practice mean marks do not differ significantly when no other factors were taken into consideration, and it shows the same results when both factors are included in the analysis. As a conclusion, education level affects knowledge and attitude marks of participants, while household income only affects attitude marks but not other categories.

## 4. Discussion

We conducted a survey to assess the level of Knowledge, Attitude, and Practice related to dengue in selected hotspot and non-hotspot areas of dengue outbreaks in Selangor. The results indicate that communities living in non-hotspot areas of dengue had better knowledge and attitude about dengue than communities living in hotspot areas, but no significant difference was found in the practice category. To the best of our knowledge, there are no previous studies conducted to compare knowledge, attitude, and practice between communities living in hotspot and non-hotspot areas in Malaysia. The findings from this study can be compared with other studies done on mosquito-borne diseases like Zika, chikungunya, and yellow fever.

Results from this survey illustrate that while the majority of respondents had average knowledge on dengue, they were not clear about dengue treatment. This suggests that providing health education about the disease is compulsory to ensure Selangor residents can understand dengue fever better, develop greater knowledge on dengue transmission, and provide more helpful attitudes towards dengue outbreak.

This study showed that the better the knowledge and attitude the respondents had, their risk from being infected by dengue fever was lower. A study by Wan Rozita et al. reported that respondents of a KAP questionnaire who were infected by dengue fever or had family members infected by dengue showed better knowledge, attitude, and practice compared to those who did not [10]. Those people with a better knowledge and attitude score were more likely to take extra precautions to prevent dengue infections [11]. It is also believed that whenever a dengue outbreak happens in an area, people around the area will be more cautious and improve their dengue vector control practice. This was proven by a study done in Singapore where while knowledge remained the same, attitude and practices on dengue improved significantly among primary care physicians after a large epidemic happened [12]. However, in this study, even though dengue-infected more people in hotspot than non-hotspot areas, the knowledge and attitude of people living in the dengue outbreak areas did not seem to improve. 

People living in non-hotspot areas in this study had higher average household income than people in hotspot areas. Low education level, low socioeconomic status, and low knowledge regarding dengue fever are reported to be associated with a poor attitude towards dengue prevention program [13]. Since dengue knowledge and attitude were highly associated with dengue prevention practice, educational campaigns should be targeted for those with lower income and education [14]. For example, a study done among people who earn less than US81 per month showed that only 28.7% of the participants have a good attitude towards dengue vaccination [15].

Respondents from hotspot areas had a lower socioeconomic class than non-hotspot areas in Selangor, on average. Among people with a lower socioeconomic class, the knowledge of dengue is still inadequate, and knowledge is reported to have a significant association with education and socioeconomic status [16]. A higher percentage of communities in Puducherry, India thought that *Aedes* breed in drains and garbage rather than clean water, which showed the lack of knowledge on dengue epidemiology, and this needs urgent action by the authority in charge to give in depth public education on dengue to the people [15]. People with less knowledge on dengue transmission rarely perform control measures on dengue outbreak prevention, such as covering water containers with lids, change water containers weekly, use of fish to eat larvae, and changing water in small vases and potted plants, and this issue should be emphasized more by health personnel and village health volunteers [11]. 

According to a study done by Chen mean larval numbers of *Aedes aegypti* and *Aedes albopictus* were found significantly higher in hotspot areas, with *Aedes aegypti* scored a higher population than the latter [16]. Families with a smaller household, a more comfortable house and children take more preventive and protective measures against vector control to ensure their house and surroundings are clear from *Aedes* breeding sites. They also have more resources to keep the mosquitoes away from their place [11].

People with or without education still believe that government is more responsible for preventing dengue rather than taking the preventive measures by themselves [17]. Good local government and community partnerships help to promote successful dengue prevention, which leads to lower household risk behavior, reduced environmental risk, and effects on mosquito numbers [18]. A study by Affendi proved that knowledge is highly associated with dengue preventive behavior, provided that self-efficacy of the people is strong [19]. This demonstrates that giving knowledge, as well as influencing people’s self-efficacy, is important while giving health education, to ensure successful preventive dengue programs.

## 5. Conclusions

Communities living in non-hotspot areas had better knowledge and attitude towards dengue which caused them to have better awareness about dengue outbreaks and helped them in cooperating each other effectively in managing the cleanliness of their surrounding environment. Having basic knowledge and a good attitude towards dengue is important to decrease the risk of exposure to *Aedes* mosquitoes which consequently lowers the risk of them becoming infected with the dengue virus. Meanwhile, communities living in hotspot areas had poorer knowledge and attitude towards dengue which might make them ignore their individual roles to avoid dengue infection. This caused them to have a higher risk of exposure to dengue outbreaks as they showed less cooperation in managing the cleanliness of their areas. This study also showed that people with a higher educational background and higher household income had better knowledge and attitude towards dengue outbreaks. Public health education, together with hygiene inspection, should be done more frequently with people with a low educational level and low household income especially in hotspot areas, to educate and remind them more effectively about the danger of dengue outbreaks, and to help lower the number of dengue cases in their areas. Education about dengue-related practice should also be highlighted in all areas to help people to have more understanding and play their roles in fighting dengue outbreak effectively. Intervention studies may also be done to ascertain the difference in knowledge, attitude, and practice towards dengue before and after receiving health education to determine the effect of giving education about dengue outbreaks in both communities. Future studies may be done in other states in Malaysia to validate the result of this study.

## Figures and Tables

**Table 1:** Demographic information.

Variables	Hotspot (%)	Non-Hotspot (%)
**Gender**		
Male	73 (35.6)	52 (25.9)
Female	132 (64.4)	149 (74.1)
**Race**		
Chinese	24 (11.7)	14 (7.0)
India	29 (14.2)	13 (6.5)
Malay	152 (74.1)	170 (84.5)
Others	0 (0)	4 (2.0)
**Household income**		
<RM1500	55 (26.8)	24 (11.9)
RM1501–RM3000	77 (38.1)	44 (21.9)
RM3001–RM5000	62 (30.2)	71 (35.3)
RM5001–RM7500	9 (3.9)	41 (20.4)
>RM7500	2 (1.0)	21 (10.5)
**Housing type**		
Flat	111 (54.1)	26 (12.9)
Condominium	0 (0)	11 (5.5)
Terrace	94 (45.9)	164 (81.6)
**Education level**		
UPSR (primary)	9 (4.4)	0 (0)
PMR (lower secondary)	9 (4.4)	8 (4.0)
SPM (high secondary)	108 (52.7)	57 (28.3)
STPM (A level/equivalent)	17 (8.3)	11 (5.4)
Diploma	31 (15.1)	29 (14.4)
Degree	30 (14.6)	96 (47.9)
Others	1 (0.5)	0 (0)
**Infected by dengue before**		
No	176 (83.9)	189 (94.0)
Yes	29 (16.1)	12 (6.0)

**Table 2:** Knowledge of Respondents’ Towards Dengue Fever.

Knowledge Items	Hotspot		Non-Hotspot	
	Correct Answer N (%)	Incorrect Answer N (%)	Correct Answer N (%)	Incorrect Answer N (%)
Dengue is spread by *Aedes aegypti* or *Aedes albopictus*	183 (89.3)	22 (10.7)	162 (80.4)	39 (19.4)
A person can get dengue more than once	146 (72.7)	59 (27.3)	188 (93.5)	13 (6.5)
Dengue fever is an infectious disease	97 (47.3)	108 (52.7)	76 (37.8)	125 (62.2)
Dengue fever is a severe flu disease that can affect babies, kids, and adults	75 (36.6)	130 (63.4)	74 (36.8)	127 (63.2)
Dengue outbreak only happens during rainy season	40 (19.5)	165 (80.5)	75 (37.8)	125 (62.2)
Symptoms of dengue include:				
High fever	193 (94.1)	12 (5.9)	186 (92.5)	15 (7.5)
Cough	107 (52.2)	98 (47.8)	83 (41.3)	118 (58.7)
Joint, muscle, bone pain	176 (85.9)	29 (14.1)	186 (92.5)	15 (7.5)
Pain behind eyes	108 (52.7)	97 (47.3)	125 (62.2)	76 (37.8)
Vomiting	164 (80.0)	41 (20.0)	165 (82.1)	36 (17.9)
Loss of appetite	144 (70.2)	61 (29.8)	161 (80.1)	40 (19.9)
Rashes	179 (87.3)	26 (12.7)	166 (82.6)	35 (17.4)
Headache	163 (79.5)	42 (20.5)	149 (74.1)	52 (25.9)
*Aedes* only spread dengue virus during the day	91 (44.4)	114 (55.6)	120 (59.7)	81 (40.3)
*Aedes* reproduce in dirty water	84 (41.0)	121 (59.0)	106 (52.7)	95 (47.3)
*Aedes* reproduce in clean water found in old tires, rubbish bins, and flower pots	193 (94.1)	12 (5.9)	191 (95.0)	10 (5.0)
Dengue virus spread among humans via infected female *Aedes* bites	163 (79.5)	42 (20.5)	164 (81.6)	37 (18.4)
The only way to combat dengue is to fight *Aedes* mosquitoes	15 (7.3)	190 (92.7)	31 (15.4)	170 (84.6)
No treatment available for dengue fever	52 (25.4)	153 (74.6)	68 (33.8)	133 (66.2)
Paracetamol is very effective to fight dengue fever	133 (64.9)	72 (35.1)	109 (54.2)	92 (45.8)
Larvae killer can help in killing *Aedes* larvae	160 (78.0)	45 (22.0)	195 (97.0)	6 (3.0)
Water containers and tanks without lids should be cleaned every 7 days	174 (84.9)	31 (15.1)	172 (85.6)	29 (14.4)
Insecticides can kill adult *Aedes* mosquitoes	143 (69.8)	62 (30.2)	143 (71.1)	58 (28.9)

**Table 3:** Attitude of Respondents’ Towards Dengue Fever.

Knowledge Items	Hotspot		Non-Hotspot	
	Agree N (%)	Disagree N (%)	Agree N (%)	Disagree N (%)
Dengue fever cannot be prevented	51 (24.9)	154 (75.1)	20 (10.0)	181 (90.0)
Dengue fever cannot be treated	28 (13.7)	177 (86.3)	27 (13.4)	174 (86.6)
Only healthcare workers and volunteers are responsible for clearing *Aedes* mosquitoes breeding sites	50 (24.3)	155 (75.7)	32 (15.9)	169 (84.1)
Killing *Aedes* mosquitoes is the only way to prevent dengue	183 (89.3)	22 (10.7)	143 (71.1)	58 (28.9)
Fogging is enough to avoid dengue	94 (45.9)	111 (54.1)	71 (35.3)	130 (64.7)
Everybody has the probability to be infected by dengue	187 (91.2)	18 (8.8)	178 (88.6)	23 (11.4)
If I have dengue symptoms, I will quickly see a doctor	198 (96.6)	7 (3.4)	191 (95.0)	10 (5.0)
I am so afraid to be infected by dengue	198 (95.1)	7 (4.9)	168 (83.6)	33 (16.4)
A person cannot get dengue twice	67 (32.7)	138 (67.3)	26 (12.9)	175 (87.1)
I will not visit dengue patient in hospital	56 (27.3)	149 (72.7)	64 (31.8)	137 (68.2)
All dengue patients have the chance to heal after infected by dengue	180 (87.8)	25 (15.2)	149 (74.1)	52 (25.9)
Killing *Aedes* mosquitoes breeding sites is wasting time and hard to do	53 (25.9)	152 (74.1)	15 (7.5)	186 (92.5)
Healthy people will never get dengue	29 (14.1)	176 (85.9)	32 (15.9)	169 (84.1)
Using mosquito net can prevent dengue	133 (64.9)	72 (35.1)	165 (82.1)	36 (17.9)
You are an important person to fight dengue spread	178 (86.8)	27 (13.2)	199 (99.0)	2 (1.0)

**Table 4:** Practice of Respondents’ Towards Dengue Vector Control.

Practice Items		Hotspot		Non-Hotspot		
	Yes N (%)	No N (%)	Not Relevant N (%)	Yes N (%)	No N (%)	Not Relevant N (%)
Do you close the container lid quickly after using it?	193 (94.2)	6 (2.9)	6 (2.9)	181 (90.0)	18 (9.0)	2 (1.0)
Does your house water tank have a lid?	178 (86.9)	8 (3.9)	17 (8.3)	182 (90.5)	9 (4.5)	10 (5.0)
If you see *Aedes* larvae in the water tank, what do you do to make it clear	173 (84.4)	16 (7.8)	16 (7.8)	186 (92.5)	3 (1.5)	12 (6.0)
Do you change the water in the container of your home garden every week?	102 (49.8)	25 (12.2)	78 (68.0)	134 (66.7)	23 (11.4)	44 (21.9)
Have you changed the water in your flower vase?	99 (48.3)	20 (9.8)	86 (41.9)	151 (75.1)	9 (4.5)	41 (20.4)
Have you checked for *Aedes* larvae in your vase?	82 (40.0)	37 (18.1)	86 (41.9)	161 (80.1)	3 (1.5)	37 (18.4)
Do you check for any garbage/rubbish that can block the drainage system around your house?	165 (80.5)	19 (9.3)	21 (10.2)	189 (94.0)	5 (2.5)	7 (3.5)
If yes, have you put the garbage into its bin to clear the drain	163 (79.5)	20 (9.8)	22 (10.7)	184 (91.5)	14 (7.0)	3 (1.5)
Do you use any mosquito repellent in your house?	169 (81.0)	33 (16.1)	3 (2.9)	160 (79.6)	41 (20.4)	0 (0)
Do you use mosquito net to sleep?	28 (13.7)	168 (82.0)	9 (4.3)	32 (15.9)	168 (83.6)	1 (0.5)
Do you get involved in any dengue campaign in your area?	75 (37.1)	118 (57.6)	12 (5.3)	79 (39.3)	118 (58.7)	4 (2.0)
Have you check *Aedes* larvae in your toilet tank?	150 (73.2)	47 (22.9)	8 (3.9)	146 (72.6)	47 (23.4)	8 (4.0)
Do you check and clean your house drain and roof during the rainy season?	118 (57.6)	47 (22.9)	40 (19.5)	109 (54.2)	72 (35.8)	20 (10.0)
Do you use any cream/oil/gel/bangle to avoid *Aedes* mosquitoes?	92 (44.9)	104 (50.7)	9 (4.2)	68 (33.8)	126 (62.7)	7 (3.5)
Do you believe in traditional medicine to fight dengue?	90 (43.9)	92 (44.9)	23 (11.2)	82 (39.8)	110 (54.7)	9 (5.5)

**Table 5:** Comparison of mean marks in Knowledge and Attitude between Hotspot and Non-hotspot Communities.

Variables	Hotspot (n = 206) Mean (SD)	Non-Hotspot (n = 200) Mean (SD)	Min Mark	Max Mark	Range of Mark	Mean Difference (95% CI)	T-Statistic (df)	*p* Value
Knowledge	14.55 (3.09)	15.41 (2.75)	2	22	20	0.86 (0.288, 1.43)	404	0.003
Attitude	10.33 (2.29)	11.41 (1.38)	5	15	10	1.09 (0.72, 1.45)	404	<0.001
Practice	10.47 (2.57)	10.83 (1.75)	4	15	11	0.22 (−0.07, 0.79)	404	0.101

**Table 6:** The Effect of Confounding Factors on Knowledge, Attitude, and Practice marks between Hotspot and Non-hotspot Communities.

Variables	Confounding Factors	F Statistic	*p* Value	Partial ETA Squared
Knowledge	Education Level	4.373	0.037	0.011
	Household Income	1.048	0.273	0.003
Attitude	Education Level	21.256	<0.001	0.050
	Household Income	15.996	<0.001	0.038
Practice	Education Level	1.782	0.183	0.005
	Household Income	0.492	0.483	0.001
